# Evaluating the Impact of a Preoperative Risk Management Program on Outcomes Following Total Joint Arthroplasty: A Retrospective Cohort Study

**DOI:** 10.1016/j.artd.2025.101880

**Published:** 2025-10-27

**Authors:** Chloe Dlott, Sebastian Romero, Claire A. Donnelley, Stephanie Kaszuba, Daniel Wiznia

**Affiliations:** a Department of Orthopaedic Surgery, University of California San Francisco, San Francisco, CA, USA; b Yale Department of Orthopaedics and Rehabilitation, Yale School of Medicine, New Haven, CT, USA; c Yale School of Medicine, Yale University, New Haven, CT, USA

**Keywords:** Total Joint Arthroplasty, Preoperative Optimization, Health Disparities, Social Determinants of Health, Postoperative Outcomes

## Abstract

**Background:**

Total joint arthroplasty is an effective intervention for end-stage joint disease, but carries elevated risks for patients with comorbidities and those from historically marginalized populations. Preoperative risk management programs are designed to mitigate these risks by optimizing patient health prior to surgery. This study evaluated the impact of such a program on postoperative outcomes at a single academic institution, with attention to racial and ethnic minorities and patients with public insurance.

**Methods:**

We conducted a retrospective cohort study of 2748 patients who underwent total hip or knee arthroplasty between 2019 and 2021 at a single academic institution. Of these, 1548 patients received preoperative optimization targeting modifiable risk factors such as diabetes, obesity, and anemia, while 1200 followed standard preoperative protocols. Outcomes assessed included length of stay, prosthetic joint infection, 30- and 90-day readmissions, and emergency department (ED) visits. Patients were stratified by race/ethnicity, insurance type, and American Society of Anesthesiology physical classification.

**Results:**

The optimized cohort had a higher average Charlson Comorbidity Index (1.1 vs 0.9; *P* = .01). There were no significant differences between groups in length of stay, readmissions, or overall ED visit rates. Black patients experienced higher ED utilization within 90 days postoperatively, regardless of optimization status. Medicaid patients with severe systemic disease had the highest rates of prosthetic joint infection (3.8%), and optimization was not associated with improved outcomes in this group. Patients with American Society of Anesthesiology physical classification ≥3 had increased ED visits postoperatively despite optimization.

**Conclusions:**

Preoperative risk management did not consistently improve outcomes, particularly among patients with greater comorbidity burdens or those facing socioeconomic disadvantage. These findings support the need for tailored optimization strategies that address both clinical risk and social determinants of health.

## Introduction

### Background

The number of patients undergoing total joint arthroplasty (TJA) is expected to increase in the coming decade [1]. As the frequency of TJA increases, measures to reduce complications, such as preoperative risk management programs, have become more prevalent [[2], [3], [4]]. Preoperative risk management programs address risk factors such as diabetes, obesity, and tobacco use to both reduce complications and improve postoperative outcomes, such as length of stay (LOS) and readmissions [5]. In turn, their goal is to directly reduce readmissions, reoperations, and postoperative complication rates, which may have cost-alleviating benefits. However, these programs often implement cutoffs for hemoglobin A1C (HbA1c) and body mass index (BMI) that may limit patient access to TJA [6,7], and do not account for complications surrounding the well documented influence of social determinants of health in complication likelihood [8]. In addition, disparities in outcomes of TJA persist for patients from racial and ethnic minority backgrounds, patients with lower incomes, and patients with Medicaid insurance [9]. While preoperative risk management programs are designed to improve patient outcomes following surgery, many of the published studies describing these programs do not include stratified analyses that would allow clinicians to determine the effects these programs have on patients from racial and ethnic minority backgrounds, women, patients with lower incomes, and patients with Medicaid insurance.

### Rationale

Given that many of the initial studies on preoperative risk management programs were performed with small patient samples, analyses stratified by race, ethnicity, gender, or insurance coverage were not feasible. A preoperative risk management program was implemented at our institution in 2018 and was found to reduce LOS and emergency department (ED) visits for patients undergoing TJA [10]. This study analyzed data from a small sample (n = 104) of patients who were participating in the program and found that among the 104 patients analyzed, those in the optimized cohort had lower LOS's and fewer ED visits at 30 and 90 days [10]. However, as the program has been active since 2018, more data was now available for analysis. The management program and its benefits were internally, not externally, validated. In order to better understand the effects of preoperative risk management programs on health-care disparities in TJA, this study sought to answer the following questions: (1) Does a preoperative risk management program improve postoperative outcomes (including LOS, readmissions, and ED visits) for patients who participated in the preoperative risk management program compared to those who did not? (2) Does a preoperative risk management program improve postoperative outcomes (including LOS, readmissions, and ED visits) for patients who identify as Black or Hispanic/Latino? (3) Does a preoperative risk management program improve postoperative outcomes for patients who are insured by Medicaid or Medicare? and (4) Does a preoperative risk management program improve postoperative outcomes for patients with multiple comorbidities?

## Material and methods

### Study design and setting

This study utilized a retrospective cohort design to compare patients who participated in the preoperative risk management programs (referred to as optimized patients) to those patients who did not participate in the program (referred to as standard preoperative or nonoptimized patients). Patients who received either a total hip arthroplasty (THA) or total knee arthroplasty (TKA) at a hospital at a single academic institution with 2 primary centers between January 2019 and December 2021 were included in the dataset. Patients from March 2020-December 2020 were excluded to limit any effects from the COVID-19 pandemic. Patients who underwent bilateral TJA, a revision total joint replacement or a THA for a hip fracture were excluded. A total of 11 surgeons participated in the preoperative optimization protocol and their patient data were included in this analysis.

This study was retrospective and analyzed patients in the preoperative risk management program dependent on when a physician themselves began participating in the program. Due to tight nurse staffing levels in the preoperative risk management program, there were not enough nursing resources to support all the surgeons. Therefore, some physicians were initially not supported by the program, so their patients were not included in the preoperative risk management program. These patients (in the standard preoperative group / no preoptimization) received preoperative care from a surgeon not yet participating in the optimization program. Physicians joined the preoperative risk management program as the program expanded over the years, as nursing resources became available to support more patients. This study did not have a coordinator who assigned patients to cohorts. Instead, patients were assigned to the preoperative risk management program only when adequate nursing resources were available to support their surgeon. Patients were not chosen to participate in the program based on their comorbidities or risk factors, and patients could not opt into or out of the program when assigned to it. Patients were not randomized into the preoptimization or nonpreoptimization cohorts by the study administrators.

### Description of experiment, treatment, or surgery

Patients participating in the preoperative risk management program were managed using established institutional protocols to address risk factors such as diabetes, obesity, substance use, smoking, malnutrition, dental care, and anemia. Nurses conducted telehealth consults with patients to improve these risk factors, as well as coordinate with the patients' primary care teams and specialists. More specifically, the preoperative risk management process focused on screening and intervening in 16 categories of patient risk factors before THA and TKA. The screening addressed allergies, significant medical and surgical history, infection risks (eg, methicillin-sensitive *Staphylococcus aureus*, methicillin-resistant *Staphylococcus aureus*, HIV, hepatitis C), smoking status, obesity (BMI >38 kg/m^2^), malnutrition (BMI <20 kg/m^2^ or albumin <3.5 g/dL), cardiovascular risks (eg, known CAD, diabetes on insulin), venous thromboembolism risks (eg, history of deep vein thrombosis or pulmonary embolism), neurocognitive and substance dependencies (eg, alcohol, narcotics), diabetes (HbA1c > 8%), anemia (hemoglobin thresholds), systemic steroid use, obstructive sleep apnea (STOP-Bang score ≥4), and the availability of social support [10]. Intervention protocols included referring patients to specialists, initiating nutrition and smoking cessation programs, addressing anemia and malnutrition, and ensuring dental care and infection risk management, among other things [10]. Screening thresholds used to trigger specific interventions within each category included HbA1c > 8% for diabetes, BMI >38 for obesity, BMI <20 or albumin <3.5 g/dL for malnutrition, and STOP-Bang score ≥4 for obstructive sleep apnea, among others. These thresholds were not used as exclusion criteria to prevent a patient from receiving surgery, they were only used to indicate that a patient should receive an intervention in the optimization program, acting as triggers for intervention [10]. The optimization protocol and its associated interventions and screening thresholds are described in Supplementary Table 1. Lastly, the standard preoperative protocol included standard laboratory testing (complete blood count, basic metabolic panel, albumin for primary TJA; with type and screen for revision TJA), anemia treatment triggers (hemoglobin <12 g/dL for women, <13 g/dL for men), nutrition optimization triggers (albumin <3.5 g/dL), HbA1c (glycated hemoglobin) measurement for diabetic patients (target <7.5%), electrocardiogram if age ≥65 or with relevant cardiovascular/metabolic comorbidities, and selective additional tests (prothrombin time/international normalized ratio, HIV, hepatitis C) based on history, according to a Preoperative Primary Care Provider Letter for arthroplasty from our institution. A methicillin-sensitive *Staphylococcus aureus*/methicillin-resistant *Staphylococcus aureus* nasal swab was routinely collected. Medication guidance included continuing most daily prescriptions, holding herbal supplements 1 week prior to surgery, and anticoagulant bridging when indicated. Abnormal findings prompted referral back to the patient's primary care provider or appropriate specialist for optimization prior to surgery. Patients referred to the optimization program were managed using institutional protocols, but compliance with recommendations and achievement of target thresholds (eg, weight loss, glycemic control) were not consistently confirmed before surgery. Participation indicated referral to the program rather than documented attainment of optimized levels.

### Variables, outcome measures, data sources and bias

Sociodemographic variables including race, ethnicity, gender, insurance provider, and American Society of Anesthesiology physical classification (ASA) rating were collected for all participating patients. Outcomes included LOS, 30-day readmissions, 90-day readmissions, 30-day ED visits, and 90-day ED visits.

### Data analyses

Data normality testing was evaluated via the Shapiro–Wilk test. Pearson's chi-squared was used on univariate analysis for normally distributed data. A Wilcoxon rank-sum (Mann–Whitney) test was used for nonparametric data. Putative variables were tested using bivariate logistic regression. Multivariable models to assess for data confounding and effect modification based on preoperative optimization were developed using hierarchical simplification with log-likelihood ratio tests and assessed for goodness of fit with Akaike information criterion and Hosmer–Lemeshow discrimination. Poisson modeling was used for multivariate analysis with heteroskedasticity. Variance-covariance matrix of estimators was assessed using variance-covariance matrix of the estimators (robust). A *P* value of 0.05 was established for statistical significance. To account for baseline differences between groups, propensity score weighting was used prior to multivariate analysis. The propensity score model included age, sex, race/ethnicity, Charlson Comorbidity Index (CCI), insurance type, ASA classification, procedure type (THA vs TKA), and treatment site. Weighted analyses were used in subsequent regression models assessing outcomes. All analyses were conducted with use of STATA MP version 16 (StataCorp).

### Ethical approval

This study was conducted in compliance with regulatory and ethical guidelines, was reviewed by the Institutional Review Board at our institution, and determined exempt (IRB #2000030656).

### Demographics, description of study population

A total of 2748 patients were included, 1200 underwent standard preoperative protocols, which did not include optimization, and 1548 underwent the preoperative optimization protocol (Table 1). Patients were majority women (61% in optimized group, 58% in standard group), were significantly older in the standard group (mean age 64.8 years in standard group, and 63.4 years in optimized group, *P* < .01), were majority white (74% optimized, 77% standardized group), and non-Hispanic (90% both groups), and had no significant between-group differences in insurance, operative surgeon, or procedure performed (52% THA in optimized and 47% THA in standard preop protocol) (Table 1). Patients in the preoperative optimization group scored significantly higher on the CCI at mean of 1.1 compared to their standard preoperative cohort at mean of 0.9 (*P* = .01). No significant differences were noted between groups in terms of ASA rating (Table 1) [11,12]. Significantly more patients underwent preoperative optimization at Center 2 as compared to Center 1, 75% at Center 2 vs 25% at Center 1 (*P* < .01) (Table 1).

**Table 1:** Demographic and background variables.

Variable	Preoperative optimization	Standard preoperative	*P* value	Test
**N**	1548	1200		
Gender - Female, n (%)	944 (61)	699 (58)	.15	T-test
Age, mean (range)	63.6 (19 - 94)	64.8 (21-95)	**<.01**	Mann–Whitney
Race, n (%)			**.04**	Mann–Whitney
Black	264 (17)	180 (15)		
White	1139 (74)	924 (77)		
Other/unknown	145 (9)	96 (8)		
Ethnicity, n (%)			.88	Mann–Whitney
Non-Hispanic	1392 (90)	1081 (90)		
Hispanic/Latino	139 (9)	108 (9)		
Unknown	17 (1)	11 (1)		
CCI Index^a^, mean (range)	1.1 (0 – 9)	0.9 (0 – 9)	**.01**	Mann–Whitney
ASA Rating, n (%)			.65	Mann–Whitney
Healthy	14 (0.9)	17 (1.4)		
Mild systemic disease	606 (39)	450 (38)		
Severe systemic disease	909 (59)	719 (60)		
Incapacitating disease	19 (1.2)	14 (1.2)		
Insurance, n (%)			.73	Mann–Whitney
Medicaid	161 (10)	191 (16)		
Medicare	409 (26)	525 (44)		
Private	186 (12)	267 (22)		
Worker’s compensation	11 (0.7)	7 (0.6)		
Unknown	403 (26)	532 (44)		
Government	22 (1.4)	18 (1.5)		
Other	8 (0.5)	8 (0.7)		
Primary Procedure, n (%)			.88	Mann–Whitney
THA	807 (52)	565 (47)		
TKA	741 (48)	635 (53)		
Delivery Network, n (%)			**<.01**	Fishers Chi^2^
Center 1	714 (60)	390 (25)		
Center 2	486 (40)	1158 (75)		
Surgeon, n (%)			.47	Mann–Whitney
Surgeon 1	187 (12)	130 (11)		
Surgeon 2	86 (6)	41 (3)		
Surgeon 3	297 (19)	72 (6)		
Surgeon 4	63 (4)	267 (22)		
Surgeon 5	162 (10)	93 (8)		
Surgeon 6	115 (7)	271 (23)		
Surgeon 7	54 (4)	109 (9)		
Surgeon 8	365 (24)	20 (2)		
Surgeon 9	24 (2)	53 (4)		
Surgeon 10	115 (7)	96 (8)		
Surgeon 11	80 (5)	48 (4)		

Bold values indicate statistical significance.

a A higher CCI score indicates a sicker patient.

## Results

### Comparison of postoperative outcomes between optimized and nonoptimized patients

On univariate analysis, there were no significant differences between patients who participated in preoperative optimization and those who did not on hospital readmission at 7, 30, or 90 days, ED visits at 30 or 90 days, prosthetic joint infection (PJI), need for transfusion, or LOS (*P* > .05; Table 2).

**Table 2:** Univariate analysis of outcome variables.

Variable	Preoperative optimization	Standard preoperative	*P* value	Test
N	1548	1200		
Hospital Readmission, n (%)				Mann–Whitney
7 d	18 (1)	15 (1)	.84	
30 d	85 (5)	60 (5)	.57	
90 d	124 (8)	89 (7)	.56	
PJI, n (%)	20 (1)	12 (1)	.48	Mann–Whitney
Transfusion, n (%)	28 (2)	33 (3)	.09	
LOS Days, median (range)	2 (1-36)	2 (1-51)	.62	Mann–Whitney
ED Visit, n (%)				Mann–Whitney
30 d	188 (12)	125 (10)	.16	
90 d	266 (17)	180 (15)	.12	

### Postoperative outcomes among Black and Hispanic/Latino patients

**Figure 1:**
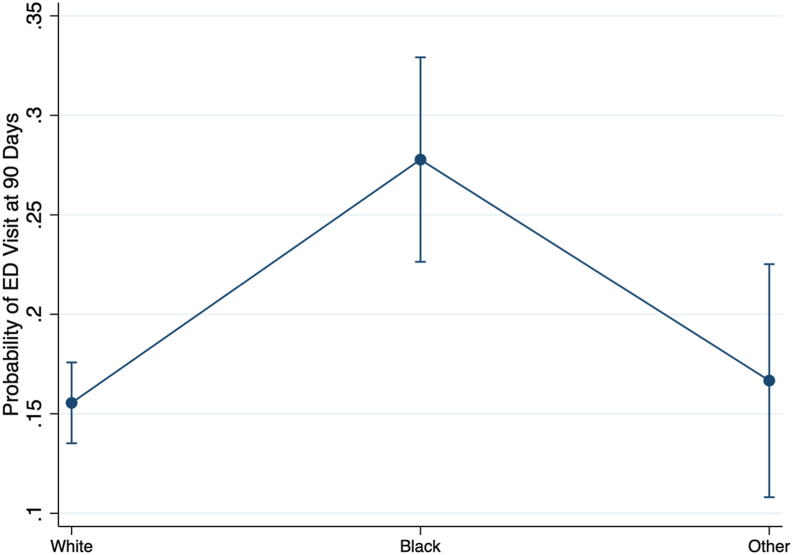
Probability of ED visit at 90 days postoperation based on race. Marginal analysis probability of visiting an ED 90 days postoperation based on race. Marginal analysis utilizing multivariate regression, in this case accounting for procedure type, surgeon, delivery network, and preoperative optimization.

The preoperative risk management program did not significantly improve postoperative outcomes, including LOS, readmissions, and ED visits, for patients who identified as Black or Hispanic/Latino (Table 4, Table 6, Table 7, and Fig. 1). Black patients had a higher probability of ED visits within 90 days postoperation compared to White patients, with marginal probabilities of 0.28 vs 0.16, respectively. The multivariate regression analysis demonstrated that Black patients had significantly more ED visits (*P* < .05). No significant differences in LOS or readmissions were observed between the optimized and standard care groups for these racial and ethnic minorities.

**Table 3:** ED presentation for patients with ASA 3 and ASA 4.

Variable	Preoperative optimization	Standard preoperative	*P* value	Test
N	928	733		
ED Visit, n (%)				Mann–Whitney
30 d	138 (15)	83 (11)	**.03**	
90 d	200 (22)	121 (16)	**<.01**	

Bold values indicate statistical significance.

**Table 4:** Multivariate regression of PJI with respect to delivery network, race, surgeon, procedure type, insurance, preoperative optimization, and ASA rating.

Variable	Impact	95% CI	*P* value
Delivery Network			
Center 2	−0.27	−1.89, 1.35	.75
Race			
Black	0.70	−0.10, 1.50	.08
Other	0.67	−0.37, 1.70	.21
Surgeon			
Surgeon 2	−15.61	−16.43, −14.78	**<.00**
Surgeon 3	0.12	−0.85, 1.1	.81
Surgeon 4	−15.58	−17.53, −13.63	**<.00**
Surgeon 5	−1.65	−2.50, 1.97	.11
Surgeon 6	−0.26	1.04, 1.24	.82
Surgeon 7	−1.27	−4.02, 1.48	.36
Surgeon 8	−0.14	−1.19, 0.90	.78
Surgeon 9	−15.32	−17.31, −13.32	**<.00**
Surgeon 10	0.38	−1.27, 2.04	.65
Surgeon 11	−1.02	−3.12, 1.08	.34
Procedure Type			
TKA	−0.97	−1.74, −0.20	**.01**
Insurance			
Medicare	−0.33	−1.11, 0.44	.40
Private	−1.19	−2.42, 0.38	.06
Workers Compensation	−15.54	−16.55, −14.52	**<.00**
Unknown	−1.15	−2.13, −0.17	**.02**
ASA Rating			
Severe Systemic Disease	1.47	0.41, 2.52	**<.00**
Preoperative Optimization	0.06	−0.68, 0.81	.87

This multivariate analysis used the outcome variable of PJI when looking at delivery network, race, surgeon, procedure type, insurance, and ASA rating including patients with mild systemic disease and severe systemic disease, or ASA ratings 2 and 3. Base case Center 1, standard preoperative optimization, White race, Surgeon 1, THA, Medicaid insurance, and ASA rating of 2, or mild systemic disease.

Bold values indicate statistical significance.

**Table 5:** Marginal probability of PJI by ASA rating and insurance type accounting for race, primary procedure, and preoperative optimization.

Variable	Probability of PJI	P value	95 % CI
Insurance/ ASA Rating			
Medicaid/ mild systemic disease	1.0%	.08	−0.01, 0.02
Medicaid/ severe systemic disease	3.8%	**< .01**	0.01, 0.06
Medicare/ mild systemic disease	0.5%	.06	−0.002, 0.01
Medicare/ severe systemic disease	2.1%	<**.01**	0.01, 0.03
Private insurance/ mild systemic disease	0.2%	.21	−0.001, 0.006
Private insurance/ severe systemic disease	1.1%	.08	−0.001, 0.02
Workers compensation/ mild systemic disease	0%	.21	−0.002, 0.002
Workers compensation/ severe systemic disease	0.004%	**.01**	0.001, 0.006
Unknown/ mild systemic disease	0.2%	.11	−0.001, 0.004
Unknown severe systemic disease	0.6%	.05	−0.001, 0.02

Bold values indicate statistical significance.

**Table 6:** Multivariate regression of ED visit at 90 d with respect to delivery network, race, insurance, preoperative optimization, CCI Index, age, and ASA rating.

Variable	Coef.	95% CI	*P* value
Delivery Network			
Center 2	0.30	0.02, 0.59	**.04**
Race			
Black	0.37	0.14, 0.61	**<.01**
Other	−0.07	−0.44, 0.30	.71
Age	−0.002	−0.013, 0.01	.65
Insurance			
Medicare	−0.16	−0.48, 0.15	.30
Private	−0.71	−1.06, −0.35	**<.00**
Workers compensation	−1.29	−3.20, 0.63	.19
Unknown	−0.50	−0.99, −0.02	**.04**
CCI Index	0.10	0.06, 0.15	**<.00**
ASA Rating			
Mild systemic disease	−0.18	−1.47, 1.10	.78
Severe systemic disease	0.15	−1.13, 1.44	.82
Incapacitating disease	0.56	−0.83, 1.94	.43
Preoperative optimization	0.06	−0.20, 0.32	.64

This multivariate analysis used the outcome variable of ED visits at 90 days postoperatively when looking at delivery network, race, insurance, CCI and ASA rating. Base case Center 1, standard preoperative optimization, White race, THA, and Medicaid insurance.

Bold values indicate statistical significance.

**Table 7:** Margin analysis probability of ED visit at 90 d with respect to race.

Variable	Margin	95% CI	*P* value
Race			
White	0.16	0.14, 0.18	**<.00**
Black	0.28	0.23, 0.33	**<.00**
Other	0.17	0.11, 0.23	**<.00**

This margin analysis probability used the outcome variable of ED visits at 90 days postoperatively when looking at race.

Bold values indicate statistical significance.

### Postoperative outcomes among patients insured by Medicaid or Medicare

**Figure 2:**
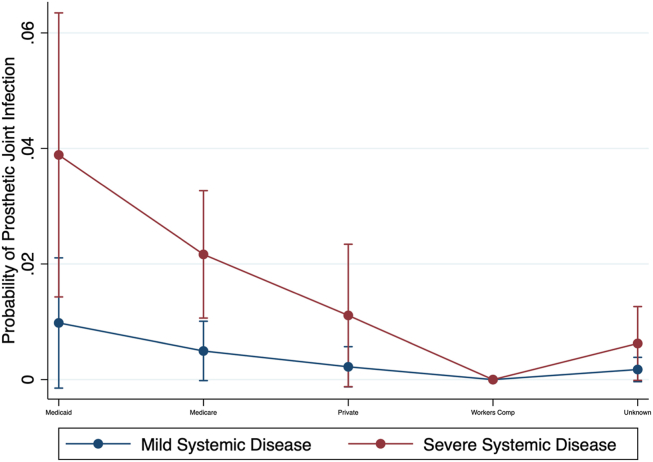
Probability of PJI by insurance type and ASA classification.marginal analysis probability of PJI based on ASA Rating and Insurance status for patients of ASA Rating 2 or 3, meaning mild or severe systemic disease respectively. Marginal analysis utilizing multivariate regression, in this case accounting for Race, Procedure Type, Surgeon, Delivery Network, and Preoperative Optimization.

The preoperative risk management program did not significantly improve postoperative outcomes for patients insured by Medicaid or Medicare (Table 4, Table 6). The multivariate regression analysis indicated that Medicaid patients had higher rates of ED visits within 90 days postoperation compared to those with private insurance (*P* < .05). On marginal analysis, patients with Medicaid insurance coverage and severe systemic disease had the highest rates of PJI (Table 5, Fig. 2) at 3.8% compared to those with private insurance and mild systemic disease (0.2%) (*P* < .05).

### Postoperative outcomes in patients with multiple comorbidities

For patients with ASA 3 or ASA 4 scores, patients who underwent preoperative optimization had significantly higher rates of 30 and 90 ED visits compared to the nonoptimized group (15% vs 11%, respectively) at 30 days (*P* = .03) and 22% vs 16% respectively at 90 days (*P* < .01) (Table 3). On multivariate analysis including delivery network, race, operative surgeon, procedure type, insurance, preoperative optimization, and ASA rating for patients with mild or severe systemic disease as classified by ASA scores 2 and 3, 3 surgeons had significantly lower rates of PJI (*P* < .01), TKA had significantly lower rate of PJI than THA (*P* = .01), and patients with severe systemic disease had significantly higher rates of PJI than their counterparts with mild systemic disease (*P* < .01) (Table 4). Patients with higher CCI and ASA scores in the optimized group had paradoxically higher rates of postoperative complications, including ED visits (*P* < .05).

## Discussion

### Background and rationale

This retrospective cohort study, conducted on 2748 patients undergoing TJA at a single academic institution, provides valuable insights into the effects of preoperative risk management programs on surgical outcomes. The division of patients into those who underwent preoperative optimization (1548) and those who received standard care (1200) allowed for a comprehensive analysis of outcomes such as LOS, readmissions, and ED visits (Table 2). Previously, analysis of preoperative optimization programs had been run on small samples and at one time point [10]. Given the availability of data starting in 2019 this large dataset allowed for stratification of racial, ethnic, and health insurance differences. Preoperative risk management programs are designed to optimize patients' health prior to surgery with the goal of improving postoperative outcomes such as LOS, readmissions, and ED visits. These programs typically focus on addressing modifiable risk factors and ensuring patients are in the best possible condition before undergoing major surgical procedures. However, the effectiveness of these programs across different patient populations, particularly those who are socioeconomically disadvantaged or have multiple comorbidities, remains an area of active investigation. This study aimed to assess whether preoperative optimization can lower complication rates faced by these vulnerable groups.

### Preoperative risk management between optimized and nonoptimized patients

In this study, the comparison between optimized and nonoptimized patients undergoing TJA revealed that preoperative risk management did not consistently yield improved postoperative outcomes. Specifically, there were no significant differences in LOS, readmissions, or ED visits across the entire cohort (Table 2), nor within key demographics such as White patients (Table 4). These findings should be interpreted in the context that the optimized group had a higher CCI, indicating more complex health profiles.

### Preoperative risk management and racial/ethnic disparities

The study found that the preoperative risk management program did not significantly improve postoperative outcomes for patients who identify as Black or Hispanic/Latino. Despite undergoing optimization, Black patients had higher rates of ED visits within 90 days postoperation compared to White patients (marginal probabilities of 0.28 vs 0.16; *P* < .05). The persistent higher rates of ED visits among Black patients highlights the need for more targeted preoperative and postoperative care strategies to effectively reduce racial disparities in surgical outcomes. It has been documented that racial socioeconomic disparities affect complication likelihoods [8]. In turn, tailoring preoperative optimization programs to further address social determinants of health may improve outcomes.

### Preoperative risk management and insurance status

For patients insured by Medicaid or Medicare, the preoperative risk management program did not lead to significant improvements in postoperative outcomes. Medicaid patients, particularly those with severe systemic disease, had higher PJI rates and increased ED visit rates compared to patients with private insurance (PJI rates of 3.8% vs 0.2%; *P* < .05 for severe systemic disease). These findings indicate that despite the implementation of preoperative optimization, socioeconomic factors related to insurance status [8] continue to play a significant role in postoperative complications. Therefore, additional support mechanisms and tailored interventions for these social determinants of health are necessary to better address the needs of patients covered by Medicaid and Medicare.

### Preoperative risk management and multiple comorbidities

The preoperative risk management program did not significantly improve postoperative outcomes for patients with multiple comorbidities. Patients with higher CCI and severe ASA scores in the optimized group experienced higher rates of 30-day and 90-day ED visits (15% vs 11% for 30-day ED visits and 22% vs 16% for 90-day ED visits; *P* < .05). This suggests either that the optimization efforts may not be adequately tailored to patients with multiple comorbid conditions, or that those in the optimization cohort needed further optimization to reach the level of nonoptimized patients given their higher CCIs. Enhancing the preoperative care protocols to more effectively address the specific risks associated with high comorbidity burden is essential for improving outcomes in this high-risk population, as well. This may include addressing other social determinants of health.

### Limitations

This study has several limitations inherent to its retrospective and observational design. The biggest limitation of this study is that it is performed at a single health-care system. Encounters at outside hospitals were not recorded, which may lead to an underestimation of true utilization rates. To mitigate this, nurses conducted 30-day follow-up surveys to inquire about ED readmissions at other health-care systems and included those episodes in a patient's chart. Additionally, patients were not randomly assigned to the preoperative optimization program. Instead, enrollment was based on surgeon participation and the availability of nursing resources, which introduces selection bias. Patients could not opt into or out of the program, but differences in provider onboarding timelines and staffing constraints may have led to unmeasured variability in patient selection. In addition, although we used propensity score weighting and multivariable regression to adjust for confounding, residual confounding remains possible, particularly from unmeasured social determinants of health, health literacy, and support systems. Patients in the optimization cohort had a higher CCI, indicating more complex disease profiles in this cohort. As a result, this may have also influenced the results indicating limited improvements in postoperative outcomes for those patients enrolled in the preoperative optimization program. The patients who were sent for optimization were not chosen based on their risk factors, but were enrolled if their surgeon participated in the program. The higher CCI in the optimized cohort may indicate that the surgeons who were initially chosen for the risk management program were seeing more complex, sicker patients. Patients who were in the optimization program had greater baseline comorbidity or risk, introducing selection bias. The study also relies on administrative data, which may not capture all relevant clinical nuances and patient characteristics. During chart analysis, a large proportion of patients in both cohorts had “unknown” insurance status due to insufficient medical record data. Additionally, it was outside the scope of this paper to say how many patients were offered surgery and then had to cancel. Patient-related outcome measure scores were not collected across the study population and thus could not be incorporated into our analysis. This limits our ability to assess the patient-perceived impact of the optimization program on functional status, pain, and quality of life following arthroplasty. Educational attainment was not captured in our dataset. This represents a limitation, as patient education and health literacy may influence comprehension of surgical risks, adherence to optimization recommendations, and postoperative recovery. Lastly, the staggered implementation of the optimization program over time may introduce temporal bias. While we attempted to account for this by including calendar time and provider variables in our models, future studies using formal time-trend or interrupted time series analyses may better isolate program effects. Given the nonrandomized design and the higher baseline comorbidity burden in the optimized group, these findings should be interpreted with caution as the lack of observed benefit may in part reflect residual confounding, rather than the absence of a true effect. Future studies should take into account randomization.

## Conclusions

Ultimately, in this single-institution nonrandomized cohort, the preoperative risk management program in this study did not significantly improve postoperative outcomes for all patients, including those identifying as Black or Hispanic/Latino, those insured by Medicaid or Medicare, or those with multiple comorbidities. Persistent disparities in ED visit rates and infection rates were observed in these high-risk groups, underscoring the limitations of current preoperative optimization strategies.

The results of this study underscore the need for further targeted, rather than uniform, application of preoperative optimization strategies. It appears that customization of these programs to address the specific risks associated with different patient demographics and comorbidity profiles could enhance their effectiveness. The optimized group, characterized by a higher CCI, may have required more intensive or targeted interventions beyond the standard optimization protocols, for example. Additionally, tailoring interventions based on patient comorbidity profiles, socioeconomic factors, social determinants of health, and demographic characteristics may enhance the program's efficacy and address persistent disparities in postoperative outcomes.

To build on these findings, future research should aim to explore which elements of preoperative optimization are most effective for different patient groups. Moreover, developing adaptive preoperative management strategies that are responsive to the nuanced needs of diverse patient populations could help in minimizing postoperative complications and enhancing overall surgical outcomes. In addition, given the inherent limitations of our single-center, nonrandomized design, adequately powered multicenter randomized controlled trials are needed to validate these results and determine the true impact of preoperative optimization on surgical outcomes.

## Conflicts of interest

The authors declare there are no conflicts of interest.

For full disclosure statements refer to https://doi.org/10.1016/j.artd.2025.101880.

## CRediT authorship contribution statement

**Chloe Dlott:** Writing – review & editing, Writing – original draft, Project administration, Formal analysis, Conceptualization. **Sebastian Romero:** Writing – review & editing, Writing – original draft, Formal analysis, Conceptualization. **Claire A. Donnelley:** Writing – review & editing, Writing – original draft, Data curation. **Stephanie Kaszuba:** Writing – original draft, Data curation, Conceptualization. **Daniel Wiznia:** Writing – review & editing, Supervision, Project administration, Data curation.

## Figures and Tables

**Supplementary Table 1:** Preoperative risk management program screening criteria and interventions.

Risk factor category	Screening criteria & intervention
Allergies	Review allergy history; document in medical record.
Medical and surgical history	Review significant history; coordinate with primary care and specialists as needed.
Infection risks	Screen for MSSA, MRSA, HIV, hepatitis C; implement infection control protocols.
Smoking status	Identify current smokers; refer to smoking cessation program.
Obesity	BMI >38; refer to nutrition services for weight management.
Malnutrition	BMI <20 or albumin <3.5 g/dL; refer to nutrition services.
Cardiovascular risks	Known CAD, diabetes on insulin; refer to cardiology/endocrinology as appropriate.
Venous thromboembolism risks	History of DVT or PE; initiate risk-reduction strategies per protocol.
Neurocognitive and substance dependencies	Assess for alcohol/narcotic use; refer to support programs.
Diabetes	HbA1c > 8%; refer to endocrinology for optimization.
Anemia	Low hemoglobin per institutional thresholds; initiate anemia management protocol.
Systemic steroid use	Assess chronic steroid use; coordinate with prescribing provider for taper/management.
Obstructive sleep apnea	STOP-Bang score ≥4; refer for sleep study or perioperative precautions.
Dental care	Dental evaluation; clearance required for infection risk management.
Social support	Assess availability; coordinate with social work or care coordination services.

Categories of Risk Factors Addressed in the Preoperative Risk Management Program.

CAD, coronary artery disease; MSSA, methicillin-sensitive *Staphylococcus aureus*; MRSA, methicillin-resistant *Staphylococcus aureus*; DVT, deep vein thrombosis; PE, pulmonary embolism; STOP-Bang, Snoring, Tiredness, Observed apnea, high blood Pressure, BMI, Age, Neck circumference, Gender.

## Appendix A Supplementary data

Conflict of Interest Statement for Dlott

Conflict of Interest Statement for Donnelley

Conflict of Interest Statement for Romero

Conflict of Interest Statement for Kaszuba

Conflict of Interest Statement for Wiznia
