# Supplementary material for: Evaluating the Impact of a Preoperative Risk Management Program on Outcomes Following Total Joint Arthroplasty: A Retrospective Cohort Study
Source: Arthroplast Today. 2025 Oct 27;36:101880. doi: 10.1016/j.artd.2025.101880 (PMC12597070; doi:10.1016/j.artd.2025.101880)
Supplement: Conflict of Interest Statement for Dlott [file mmc1.pdf]

# CONFLICT OF INTEREST STATEMENT

## *American Association of Hip and Knee Surgeons*

(Adopted from the American Academy of Orthopaedic Surgeons disclosure statement)

The following form **must be filled out completely and submitted by each author (example, 6 authors, 6 forms). All items require a response. If there is no relevant disclosure for a given item, enter "*None.*"**

Manuscript Title
**Evaluating the Impact of a Preoperative Risk Management Program on Outcomes Following Total Joint Arthroplasty: A Retrospective Cohort Study**

1. Royalties from a company or supplier (The following conflicts were disclosed)

2. Speakers bureau/paid presentations for a company or supplier (The following conflicts were disclosed)

3A. Paid employee for a company or supplier (The following conflicts were disclosed)

3B. Paid consultant for a company or supplier (The following conflicts were disclosed)

3C. Unpaid consultants for a company or supplier (The following conflicts were disclosed)

4. Stock or stock options in a company or supplier (The following conflicts were disclosed)

5. Research support from a company or supplier as a Principal Investigator (The following conflicts were disclosed)

6. Other financial or material support from a company or supplier (The following conflicts were disclosed)

7. Royalties, financial or material support from publishers (The following conflicts were disclosed)

8. Medical/Orthopaedic publications editorial/governing board (The following conflicts were disclosed)

9. Board member/committee appointments for a society (The following conflicts were disclosed)

**Each author must sign AND print or type his/her name, date and submit a separate form**

In addition, one BLINDED Conflict of Interest form (no author names used) should be submitted per manuscript with all author disclosures.

| Chloe Dlott | Chloe D | 04/18/2025 |
|---|---|---|
| Author Name (Print or Type) | Author Signature | Date |
